# Case Report: Diverse cardiac and muscular phenotypes in DES c.1024A>G (p.Asn342Asp) variant: a case series with limb weakness as the initial presentation

**DOI:** 10.3389/fcvm.2025.1590306

**Published:** 2025-06-02

**Authors:** Liuyang Wang, Dongyue Yue, Zijun Chen

**Affiliations:** ^1^ Department of Cardiology, The Affiliated Yongchuan Hospital of Chongqing Medical University, Chongqing, China; ^2^ Department of Neurology, Jing’an District Central Hospital, Shanghai, China

**Keywords:** desmin, desminopathy, limb weakness, atrioventricular block, atrial fibrillation, left ventricular hypertrophy, cardiomyopathy

## Abstract

We report three patients with bilateral lower limb weakness as the initial symptom. Case 1 presented at 37 years old with bilateral lower limb weakness. The condition gradually worsened, eventually leading to wheelchair dependence. He later sought medical attention for heart failure. Echocardiography showed diffuse left ventricular dysfunction, and the electrocardiogram revealed third-degree atrioventricular block. Case 2 developed bilateral lower limb weakness at 38 years old, with milder symptoms. The main cardiac manifestation was paroxysmal atrial fibrillation. Case 3 presented at 33 years old with lower limb weakness and myalgia, with significant involvement of all four limbs. The primary cardiac finding was left ventricular hypertrophy, and the electrocardiogram showed sinus pauses. Despite the differences in clinical presentations, all three patients were diagnosed with the same DES c.1024A>G (p.Asn342Asp) variant. We discuss the possible factors contributing to the phenotypic differences. Based on the uniqueness of this pathogenic variant site, we propose recommendations for the treatment and management of desminopathy.

## 1 Introduction

Desmin, a muscle-specific intermediate filament protein, serves as a critical structural scaffold that is essential for maintaining the stability of muscle cells (1). In the OMIM database, Pathogenic variants in the desmin gene are associated with three clinically defined entities: (1) Myofibrillar Myopathy 1, characterized by progressive limb-girdle weakness, desmin-positive cytoplasmic aggregates on histopathology, and cardiac conduction (2); (2) Dilated Cardiomyopathy 1Ⅰ, predominantly manifesting as left ventricular dilation, impaired contraction, and malignant ventricular arrhythmias (3); and (3) Neurogenic Scapuloperoneal Syndrome, Kaeser Type, featuring scapuloperoneal distribution of weakness and atrophy (4). Among these, desminopathy, a rare inherited myopathy, is caused by pathogenic variants in the desmin gene (DES) (5), leading to progressive skeletal muscle weakness and a range of cardiac manifestations, including cardiac conduction disorders, arrhythmias, and cardiomyopathy (6). The DES c.1024A>G (p.Asn342Asp) variant has been identified in Dutch and Irish-German populations (1, 7–9). Despite its recognition, the full spectrum of clinical outcomes associated with this variant remains poorly understood. This case series presents, for the first time, three Chinese patients. Each patient exhibits distinct clinical manifestations, providing new insights into this complex disorder.

## 2 Case series

### 2.1 Case 1

**Figure 1:**
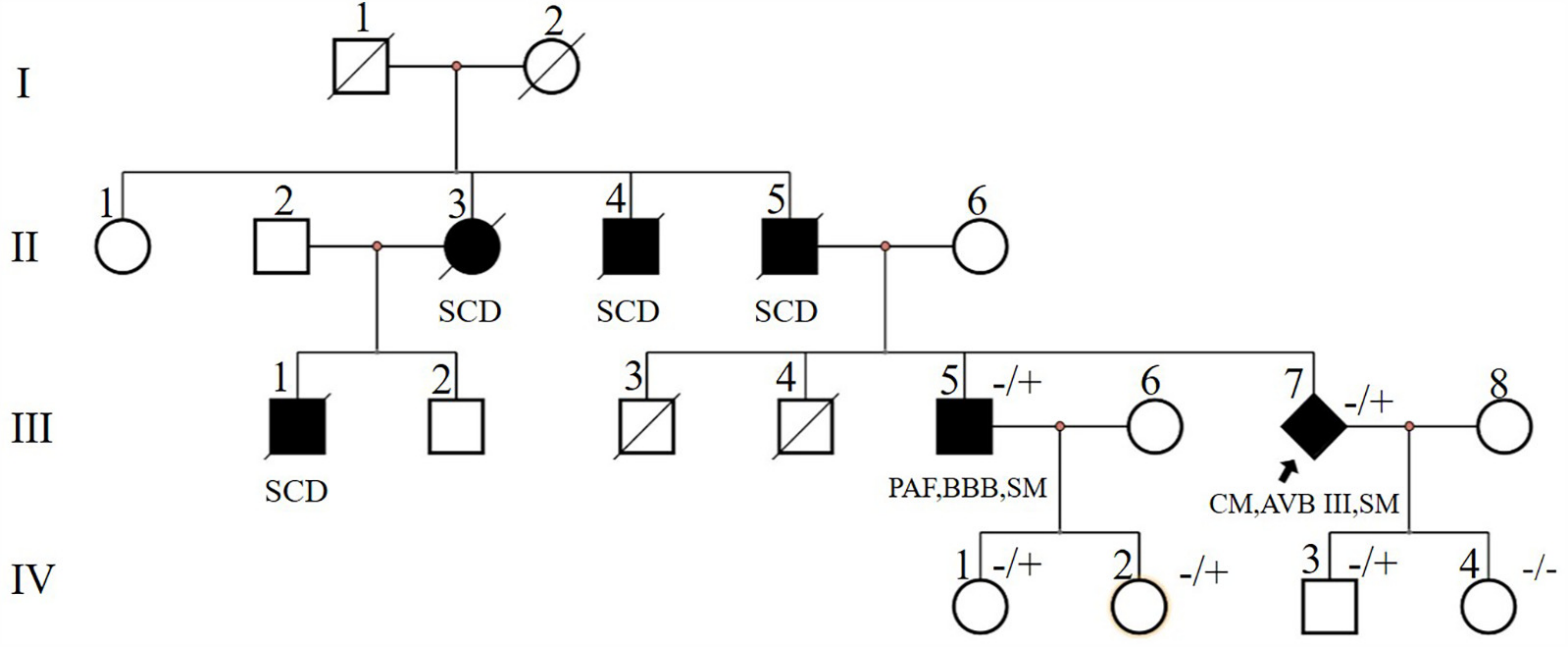
Pedigree of case1 (Ⅲ-7) and case2 (Ⅲ-5). Circles represent females, squares males, slash denotes deceased. Black filled symbols indicate individuals with clinical phenotype. PAF, paroxysmal atrial fibrillation; CM, cardiomyopathy; SCD, sudden cardiac death; AVB, atrioventricular block; BBB, bundle branch block; SM, skeletal myopathy; −/+, heterozygous allele; −/−, wild type for both DES alleles. The index patient is marked with an arrow (rhombus symbol indicate male patient).

**Figure 2:**
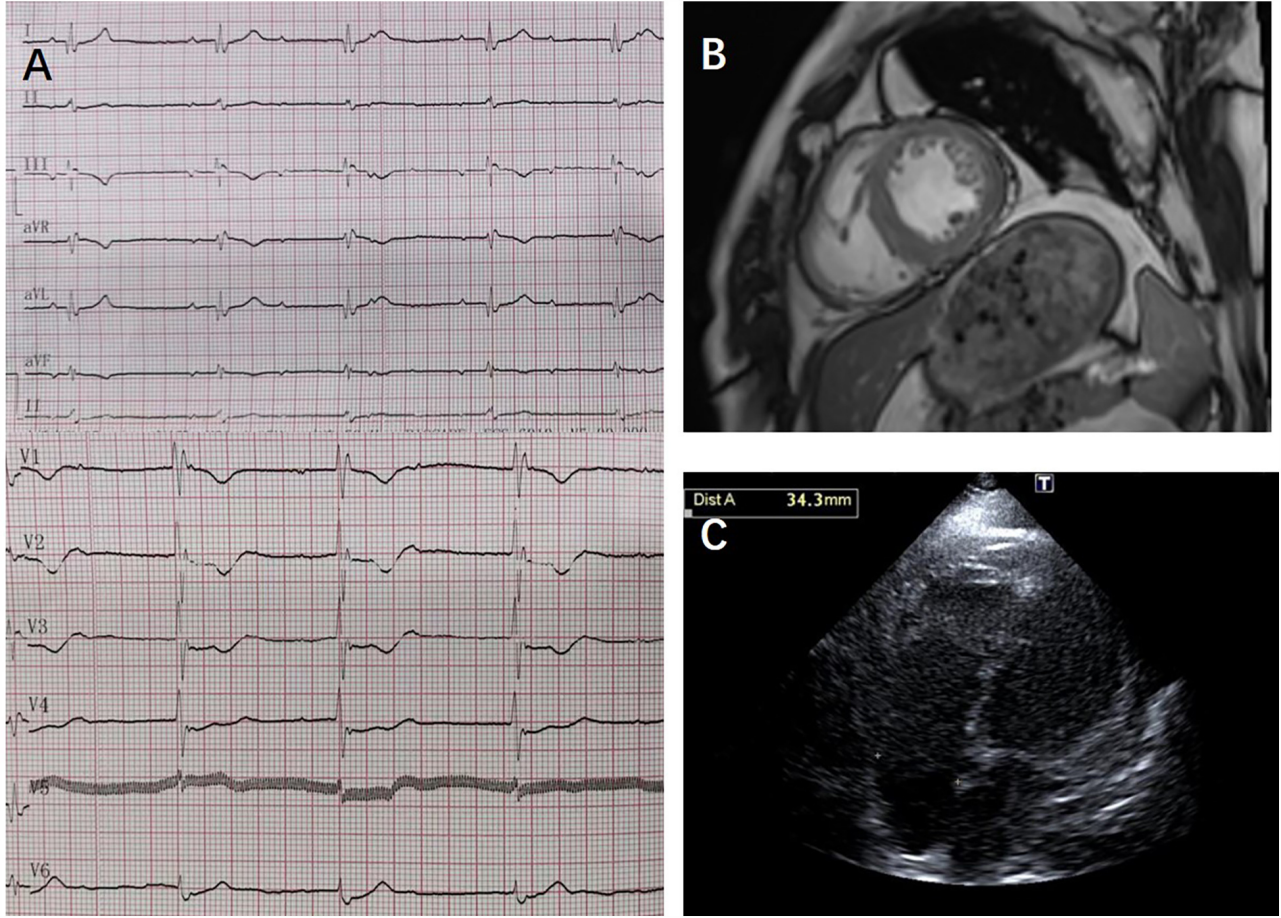
ECG, cardiac MRI, and transthoracic echocardiography results from case 1: **(A)** transthoracic echocardiography showed no ventricular or atrial dilation. **(B)** Cardiac MRI showed no perfusion or delayed enhancement abnormalities; **(C)** ECG showed third-degree atrioventricular block, junctional escape rhythm, and complete right bundle branch block.

A 50-year-old male (individual Ⅲ-7, Figure 1) presented to our hospital with paroxysmal nocturnal dyspnea accompanied by bilateral lower extremity edema. After diuretic therapy, significant atrophy of both lower limbs was observed, with muscle strength graded at 0 (MRC scale), while the upper limbs showed no significant abnormalities (grade 5, MRC scale). Serum troponin I was 0.045 ng/ml (normal reference value <0.037 ng/ml), creatine kinase MB isoenzyme was 10 ng/ml (<2.37 ng/ml), and N-terminal pro B-type natriuretic peptide (NT-proBNP) was 2,248 pg/ml (<125 pg/ml). Transthoracic echocardiography revealed diffuse left ventricular dysfunction (ejection fraction 47%) without any evidence of ventricular or atrial dilation (Figure 2A). Mild tricuspid and aortic valve regurgitation was noted. Electrocardiogram (ECG) showed sinus rhythm, first-degree atrioventricular block (AVB), complete right bundle branch block, and left anterior fascicular block. Cardiac magnetic resonance imaging (MRI) revealed no perfusion or delayed enhancement abnormalities (Figure 2B). Coronary CT angiography showed mild stenosis in the midportion of the left anterior descending artery, with no other vascular abnormalities. The patient was treated with furosemide and spironolactone after discharge.

Two years later, the patient presented with worsening dyspnea. Upon examination, muscle strength in the small finger abductor of both upper limbs was graded at 3 (MRC scale). ECG revealed third-degree AVB, junctional escape rhythm, and complete right bundle branch block (Figure 2C). Transthoracic echocardiography showed no worsening of cardiac function. Electromyography (EMG) indicated myogenic electrophysiological damage. The patient had experienced bilateral lower limb weakness and atrophy at the age of 37. His father (individual II-5, Figure 1), uncle (individual II-4, Figure 1), aunt (individual II-3, Figure 1), and older brother (individual III-1, Figure 1) also had similar symptoms. All have since passed away, but the exact cause of death is unknown. Given the involvement of both cardiac and skeletal muscles, along with a family history of similar symptoms, we suspected a hereditary muscle disease. Genetic testing revealed a DES c.1024A>G (p.Asn342Asp) variant. Based on guidelines from the American College of Medical Genetics and Genomics (ACMG), the variant was classified as pathogenic (PS4_Moderate + PM2 + PP1_Strong + PP3). Supporting evidence: PS4_Moderate: This variant has been identified in patients with desminopathy (1). PM2: The variant was not found in normal control populations in the ESP, 1,000 Genomes, or EXAC databases. PP1_Strong: The variant was found in multiple patients within the same family with myopathy and cardiomyopathy (7). PP3: Various in silico prediction tools (SIFT, MutationTaster) suggest the variant is likely to have a deleterious impact on the gene or its product.

The patient underwent permanent pacemaker implantation and was treated with diuretics (furosemide, spironolactone orally), sacubitril/valsartan 25 mg twice daily, and dapagliflozin 10 mg once daily. His symptoms of dyspnea and lower extremity edema improved, and he was discharged.

### 2.2 Case 2

After the diagnosis of desminopathy in patient III-7 (Figure 1), we conducted a systematic examination of his older brother (individual III-1, Figure 1). At 38 years old, he developed bilateral lower limb weakness and, at 53 years old, presented to our hospital's neurology department with cardiogenic cerebral embolism. He reported exertional dyspnea and palpitations. Muscle strength in his lower limbs was graded at 3 (MRC scale), while his upper limbs had normal strength (grade 5, MRC scale). ECG showed paroxysmal atrial fibrillation. Transthoracic echocardiography revealed left atrial enlargement with normal systolic function. Cardiac ultrasound showed left atrial enlargement, and creatine kinase (CK) levels were normal. NT-proBNP was elevated at 1,140 pg/ml. EMG indicated myogenic electrophysiological damage. Genetic testing revealed that he carried the familial DES c.1024A>G (p.Asn342Asp) variant. The patient was treated with rivaroxaban 20 mg once daily, losartan potassium 25 mg once daily, and dapagliflozin 10 mg once daily.

Three years later, follow-up cardiac ultrasound showed minimal change in left atrial enlargement with normal systolic function. ECG revealed first-degree atrioventricular block, complete right bundle branch block, and left anterior fascicular block. Holter monitoring showed sinus rhythm with paroxysmal atrial fibrillation, accompanied by 17 episodes of sinus pause lasting more than 2 s (the longest being 3.6 s), with no severe atrioventricular block observed. During follow-up, the patient reported mild improvement in exercise tolerance.

### 2.3 Case 3

**Figure 3:**
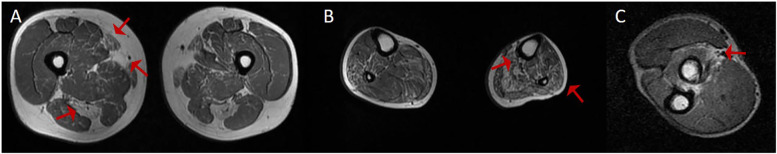
The MRI results of the upper and lower limbs in case 3. **(A)** MRI T1-weighted imaging of the thigh in another patient demonstrated selective fatty infiltration in the semitendinosus, sartorius, and gracilis muscles (red arrows). **(B)** MRI T1-weighted imaging of the lower leg revealed fatty infiltration in the fibularis and soleus muscles (red arrows). **(C)** MRI T1-weighted imaging of the forearm muscle revealed fatty infiltration (red arrows).

**Figure 4:**
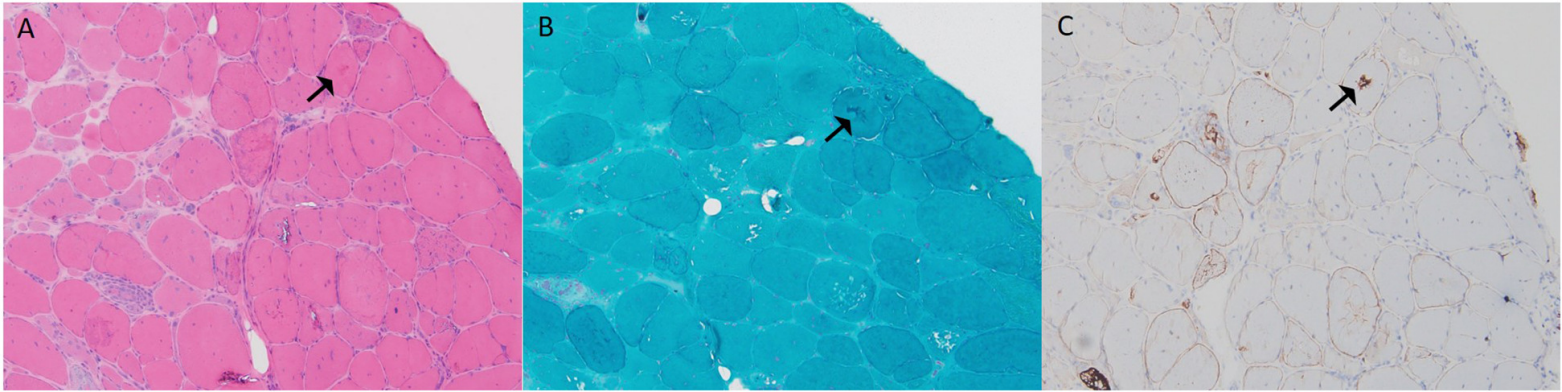
Muscle biopsy pathology results from case 3: **(A)** H&E staining (×100) showed marked variation in fiber size, irregular morphology, and numerous hypertrophic and split fibers, with purplish-red deposits in some fibers (black arrow). **(B)** MGT (×100) revealed abnormal deposits in some fibers (black arrow). **(C)** Desmin (×100) was positive (black arrow).

A 33-year-old male presented with bilateral lower limb weakness, which was subsequently accompanied by myalgia following physical activity. Muscle strength in the upper limbs was graded at 5 (MRC scale), while the left hip extensors were graded at 4, the left dorsal flexors at 4, and the plantar flexors at 3. The right plantar flexors showed grade 3 strength, and the rest of the limb muscles had normal strength. CK was elevated to 834 U/L (<170 U/L), serum troponin T was 0.164 ng/ml (<0.03 ng/ml), and NT-proBNP was 2,137 pg/ml (<125 pg/ml). Holter monitoring revealed sinus rhythm with 16 episodes of sinus pauses lasting more than 2 s (the longest being 2.4 s). Transthoracic echocardiography demonstrated left ventricular apical hypertrophy, approximately 15–16 mm, with normal systolic function. Muscle MRI of the lower limbs showed varying degrees of fat infiltration in the semitendinosus, sartorius, gracilis, peroneus, and gastrocnemius muscles (Figure 3). The MRI of the right forearm muscles showed fat infiltration in the supinator muscle (Figure 3). A biopsy of the right tibialis anterior muscle revealed significant variation in fiber size and irregular morphology, with numerous hypertrophic and split fibers. Hematoxylin and eosin (HE) staining revealed purplish-red deposits and many atrophic fibers with peripheral vacuoles. Masson's trichrome staining (MGT) showed abnormal deposits within some fibers, and desmin immunohistochemistry was positive (Figure 4). Genetic testing revealed a DES c.1024A>G (p.Asn342Asp) variant, inherited from the patient's father (who presented with a normal phenotype).

Three years later, follow-up echocardiography showed hypertrophy of the left interventricular septum, in addition to the apical hypertrophy (apex thickness 14–17 mm, interventricular septum thickness 17–18 mm), with normal systolic function.

## 3 Discussion

We report three patients carrying the DES c.1024A>G (p.Asn342Asp) variant. Despite carrying the same genetic variant, they exhibit significant differences in clinical phenotypes. Case 1 presented with bilateral lower limb weakness at the age of 37, which progressively worsened, leading to wheelchair dependency. The patient later sought medical attention due to heart failure. Echocardiography revealed diffuse left ventricular dysfunction, and the electrocardiogram showed significant cardiac conduction abnormalities, including first-degree AVB, complete right bundle branch block, and left anterior fascicular block. Over time, the conduction block worsened, and the patient eventually required implantation of a permanent dual-chamber pacemaker. In contrast, Case 2 also experienced bilateral lower limb weakness at age 38, though the severity was milder compared to Case 1. The primary cardiac manifestation was paroxysmal atrial fibrillation, without significant AVB or left ventricular dysfunction. Echocardiography indicated mild left atrial enlargement, and the patient's cardiac structure and function remained stable during follow-up. Case 3 presented with both lower limb weakness and myalgia, with significant involvement of all four limbs. The primary cardiac finding was left ventricular hypertrophy, and the electrocardiogram showed sinus pauses (up to 2.4 s), but no atrioventricular block, and normal cardiac contractile function. Case 3 was initially reported one year ago (10). During follow-up, left ventricular hypertrophy worsened, but there was no significant decline in cardiac function or severe electrophysiological abnormalities.

The cardiac involvement exhibited different patterns. On one hand, progressive conduction disturbances (e.g., in Case 1, where first-degree AVB progressed to third-degree AVB) may be associated with the disruption of intercalated disc structures caused by desmin deficiency. Brodehl et al. (11) created an induced pluripotent stem cell model expressing the p.Y122H variant and performed functional analysis. They found that this variant resulted in severe defects in filament assembly and desmin aggregation, which might contribute to AVB in patients. Variants in the DES gene can disrupt the intermediate filament network within cardiomyocytes. This affects both the structure and function of the heart muscle cells. Ultimately, it leads to heart failure (11, 12). On the other hand, left ventricular hypertrophy may reflect compensatory hypertrophy of cardiomyocytes in response to mechanical stress transmission dysfunction (13). Additionally, desmin deficiency causes cytosolic calcium overload and reduced sarcoplasmic reticulum calcium, which increases the risk of atrial fibrillation (14).

The muscle involvement pattern in desminopathy has distinct features. In the pelvic girdle, the gluteus maximus is more severely affected, while the gluteus medius and minimus are less involved. In the thigh, the semitendinosus, sartorius, and gracilis are often the first muscles affected. In the calf, fatty infiltration is more noticeable in the fibularis muscles, while the tibialis anterior and gastrocnemius are less affected. Studies show that involvement of the semitendinosus and fibularis muscles has high sensitivity for diagnosing desminopathy (15). In Case 3, his upper limb muscle strength was normal, but MRI showed early fatty infiltration and atrophy in the supinator muscle. This may help in the early diagnosis of desminopathy. Further cases are needed to confirm the supinator muscle's role as an early diagnostic marker.

The phenotypic differences caused by the p.Asn342Asp variant may arise from the interaction of multiple regulatory mechanisms. From a structural protein perspective, asparagine at position 342 is located within the 2B domain of desmin, which is crucial for the proper assembly of intermediate filaments. Disruption of the hydrogen bond network may impair the assembly of desmin-synemin heteropolymers, thereby affecting the mechanical and functional stability of the cytoskeleton (16). This structural defect may account for the differential involvement of myocardial and skeletal muscles (17). Cardiomyocytes have a higher demand for the integrity of the intermediate filament network due to sustained mechanical stress (18), while the regenerative capacity of satellite cells in skeletal muscle may partially compensate for the structural defect (19, 20). Moreover, incomplete penetrance can lead to different clinical manifestations in individuals carrying the same variant (20, 21, 27). For instance, although the father of Case 3 carries the same p.Asn342Asp variant, he did not exhibit any clinical symptoms. Environmental factors may act as modifiers of the phenotypic expression (22). Previous studies have shown that heat stress, oxidative stress, and mechanical stress can trigger desmin aggregation within the cytoplasm (22). Environmental stress factors, including physical exercise, may upregulate desmin expression and initiate or accelerate its aggregation (23). This process may be influenced by the tendency of different DES variants to form aggregates (24).

The p.Asn342Asp mutation is a founder mutation identified in the Dutch population (7). Unlike other variants in the DES gene, the distinctiveness of p.Asn342Asp lies in the fact that all affected individuals exhibit a neuromuscular phenotype. Moreover, the cardiac phenotype associated with p.Asn342Asp appears to exhibit full penetrance. Arrhythmia, conduction disorders, and cardiomyopathy are the main cardiac complications in these patients. Desminopathy is associated with a generally poor clinical outcome. A 10-year follow-up study of desminopathy patients revealed a mortality rate of 17.8%, with cardiac complications identified as the leading cause of death (25).

Currently, there is a lack of specific treatment for cardiac involvement in desminopathy patients. Our treatment approach primarily follows the guidelines for heart failure (26), and the medications used include diuretics, ARNI (angiotensin receptor-neprilysin inhibitor), ARBs (angiotensin II receptor blockers), SGLT2 inhibitors, and aldosterone receptor antagonists, which have resulted in symptom relief. For severe atrioventricular conduction block, the patient underwent implantation of a permanent pacemaker to maintain normal cardiac rhythm and function. Although no specific treatment is available at present, proactive cardiac management and treatment of arrhythmias can improve the patient's quality of life and prognosis.

## 4 Conclusion

Desminopathy exhibits clinical heterogeneity, which complicates diagnosis and treatment. In this report, we present, for the first time, the DES c.1024A>G (p.Asn342Asp) variant in a Chinese population, highlighting its diverse phenotypes. The three cases demonstrate considerable variability in the clinical manifestations of both skeletal and cardiac involvement. These manifestations range from progressive conduction abnormalities to paroxysmal atrial fibrillation and left ventricular hypertrophy. Given the lack of specific therapies for desminopathy, the management strategies in these cases, including adherence to heart failure guidelines and permanent pacemaker implantation, have provided symptom relief and improved quality of life. Further studies, including long-term follow-up and genetic investigations, are needed to better understand the mechanisms underlying phenotypic variability and to guide the development of targeted treatments for desminopathy.

## Data Availability

The original contributions presented in the study are included in the article/Supplementary Material, further inquiries can be directed to the corresponding author.
